# Search for Possible Additional Reservoirs for Human Q Fever, the Netherlands

**DOI:** 10.3201/eid1905.121489

**Published:** 2013-05

**Authors:** Hendrik I.J. Roest, Conny B. van Solt, Jeroen J.H.C. Tilburg, Corné H.W. Klaassen, Emiel K. Hovius, Frank T.F. Roest, Piet Vellema, René van den Brom, Fred G. van Zijderveld

**Affiliations:** Central Veterinary Institute, part of Wageningen UR, Lelystad, the Netherlands (H.I.J. Roest, C.B. van Solt, F.G. van Zijderveld);; Canisius Wilhelmina Hospital, Nijmegen, the Netherlands (J.J.H.C. Tilburg, C.H.W. Klaassen);; Companion Animal Hospital 't Heike, Veldhoven, the Netherlands (E.K. Hovius);; Veterinary Practice Tusken Diken en Feanen, Drogeham, the Netherlands (T.F. Roest);; Animal Health Service, Deventer, the Netherlands (P. Vellema, R. van den Brom)

**Keywords:** Q fever, *Coxiella burnetii*, bacterial typing, polymerase chain reaction, zoonoses, cats, dogs, horses, swine, ruminants, bacteria, the Netherlands

**To the Editor:** Q fever is a zoonosis caused by the bacterium *Coxiella burnetii*. The Q fever outbreak in the Netherlands affected ≈4,000 humans during 2007–2010 (*1*). In this outbreak, 1 genotype of *C. burnetii* appeared to be responsible for abortions in small ruminants and for clinical disease in humans (*2*,*3*). However, little is known about the outbreak genotype and the prevalence of *C. burnetii* in possible additional reservoirs for human Q fever (i.e., cats, dogs, horses, sheep, and cattle) in the Netherlands.

We aimed to search for possible additional reservoirs for human Q fever in the Netherlands. Placentas from 15 cats, 54 dogs, and 31 horses were collected in 2011 at 5 veterinary practices. Placentas were collected by targeted sampling at breeding facilities and during parturition with veterinary assistance. In addition, 27 ovine, 11 caprine, 16 porcine, 8 equine, and 139 bovine placentas (originating from aborting animals from throughout the Netherlands that were submitted in 2011 to investigate the abortion cause) were included in the study. Samples were stored at −20°C before testing.

DNA was extracted from the allantochorion of the placenta and analyzed as described (*2*). Samples with sufficient DNA load (cycle threshold [C_t_] value <32) were typed by using 2 multilocus variable-number tandem-repeat analyses (MLVA) genotyping methods (MLVA-12 and MLVA-6), and the multispacer sequence typing method (*3*–*5*). Two *C. burnetii* strains from the Netherlands representing the outbreak genotype (X09003262, 3345937) and the Nine Mile RSA 493 were included as reference. For prevalence calculations, the Netherlands was divided in a southern part, comprising the Q fever hot spot area of notified cases in humans and small ruminants during the 2007–2010 epidemic (*1*,*6*), and a northern part, comprising the rest of the country.

*C. burnetii* DNA was not detected in placentas from cats, goats, or pigs. *C. burnetii* DNA was detected in 4 (7% [95% CI 0.4–14.4]) of 54 canine placentas; 3 from the north and 1 from the south of the Netherlands. *C. burnetii* DNA was detected in 3 (8% [95% CI 0.0–16.1]) of 39 equine placentas, all from the north of the country, without known abortion history. *C. burnetii* DNA was detected in 7 (26% [95% CI 9.4–42.5]) of 27 ovine and in 33 (24% [95% CI 16.7–30.8]) of 139 bovine placentas. The prevalence of *C. burnetii* DNA–positive ovine and bovine placentas from the north and the south did not differ significantly.

The *C. burnetii* DNA load in the placentas from dogs (C_t_ value 37.4–38.0) and horses (C_t _value 35.4–37.4) was too low to be suitable for genotyping. Typing of 1 positive sheep sample resulted in an incomplete genotype, which is related to the outbreak genotype (sheep 192, Figure). Seven of the 33 *C. burnetii* DNA–positive bovine placentas were suitable for typing. One sample had a genotype similar to the outbreak genotype (*2*,*3*). Six other samples revealed a (partial) genotype related to bovine genotypes from the Netherlands (*2*,*5*,*7*), including a novel one. MLVA-6 and multispacer sequence typing results were consistent with the MLVA-12 results (Figure).

**Figure:**
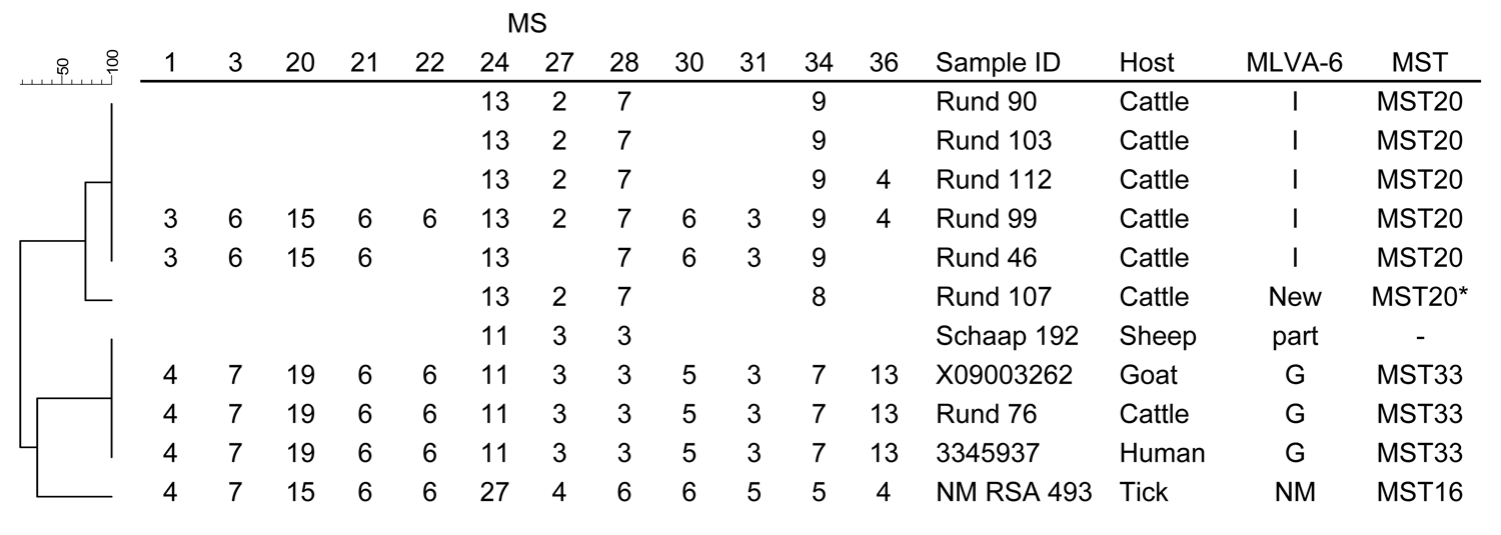
Phylogenetic tree of the genotypes of *Coxiella burnetii* from the samples of this study based on multilocus variable-number tandem-repeat analyses (MLVA) including 12 loci (MLVA-12). Repeats per locus are shown, and open spots indicate missing values. MLVA-6 are results of the analysis with 6 MLVA loci (*3*). MST are results of the analysis with multispacer sequence typing (MST) (*5*). MLVA 6 loca (MLVA-6) and MST revealed full genotypes unless stated otherwise. Two strains representing the outbreak genotype of *C. burnetii* (X09003262, 3345937) in the Netherlands and the Nine Mile (NM) RSA 493 are included as reference. MS, mini satellite; G and I, MLVA-6 genotypes of *C. burnetii* as published (3,7); MSTxx, MST genotypes as published (*5*). *Based on partial genotype; part, partial genotype. – (in MST column) indicates no results obtained. Scale bar indicates percentage similarity.

Results give no indication for major reservoirs of *C. burnetii* in cats, goats, and pigs in the Netherlands in 2011. However, the low numbers of placentas may have biased the results. Dogs and horses should be considered as reservoirs for *C. burnetii*. The detection of *C. burnetii* DNA–positive placentas in dogs and horses in the northern part of the country indicates the presence of a true reservoir rather than a spillover effect from the contaminated environment in the south. This observation is consistent with a reported seroprevalence of 13% in dogs in the Netherlands in 1992 (*1*). Until now, horses had been discussed as a risk factor in the Q fever outbreak in the Netherlands (*8*).

Prevalence data from sheep and cattle suggest that *C. burnetii* is present in placentas in 25% of the abortion cases in these species. Presence of the outbreak genotype of *C. burnetii* in sheep has been observed (*2*,*5*), indicating sheep are a reservoir for Q fever in humans. Genotyping data show a distinct genotype in 6 of the 7 cattle samples in accordance with previous work (*2*,*5*,*7*). However, the outbreak genotype was detected in 1 sample from a cow. Whether this is an incidental finding or the first observation of the outbreak genotype being transferred to the cattle population is not clear. If the latter, exposure to cattle also possibly might become a risk factor for human Q fever, in addition to goats and sheep.
